# Prognostic value of screening instrument based on the Dutch national VMS guidelines for older patients in the emergency department

**DOI:** 10.1007/s41999-020-00385-0

**Published:** 2020-09-01

**Authors:** B. M. G. Snijders, M. H. Emmelot-Vonk, E. T. D. Souwer, H. A. H. Kaasjager, F. van den Bos

**Affiliations:** 1 Department of Geriatrics, University Medical Center Utrecht, Utrecht, The Netherlands; 2 Department of Internal Medicine, Leiden University Medical Center, Leiden, The Netherlands; 3 Department of Internal Medicine, University Medical Center Utrecht, Utrecht, The Netherlands

**Keywords:** Emergency department, Older adults, Screening, Adverse outcomes, Mortality

## Abstract

**Purpose:**

It is important to identify which older patients attending the emergency department are at risk of adverse outcomes to introduce preventive interventions. This study aimed to assess the prognostic value of a shortened screening instrument based on the Dutch national Safety Management System [*Veiligheidsmanagementsysteem* (VMS)] guidelines for adverse outcomes in older emergency department patients.

**Methods:**

A cohort study was performed including patients aged 70 years or older who visited the emergency department. Adverse outcomes included hospital admission, return emergency department visits within 30 days, and 90-day mortality. The prognostic value of the VMS-score was assessed for these adverse events and, in addition, a prediction model was developed for 90-day mortality.

**Results:**

A high VMS-score was independently associated with an increased risk of hospital admission [OR 2.26 (95% CI 1.32–3.86)] and 90-day mortality [HR 2.48 (95% CI 1.31–4.71)]. The individual VMS-questions regarding history of delirium and help in activities of daily living were associated with these outcomes as well. A prediction model for 90-day mortality was developed and showed satisfactory calibration and good discrimination [AUC 0.80 (95% CI 0.72–0.87)]. A cut-off point that selected 30% of patients at the highest risk yielded a sensitivity of 67.4%, a specificity of 75.3%, a positive predictive value of 28.5%, and a negative predictive value of 94.1%.

**Conclusion:**

The shortened VMS-based screening instrument showed to be of good prognostic value for hospitalization and 90-day mortality. The prediction model for mortality showed promising results and will be further validated and optimized.

**Electronic supplementary material:**

The online version of this article (10.1007/s41999-020-00385-0) contains supplementary material, which is available to authorized users.

## Key summary points

**Aim:**

To evaluate the prognostic value of a shortened screening instrument based on the Dutch national Safety Management System [*Veiligheidsmanagementsysteem *(VMS)] guidelines for older emergency department patients.

**Findings:**

A high VMS-score is associated with elevated risks of hospitalization and 90-day mortality. A prediction model for 90-day mortality, which incorporated the VMS-score, showed promising results.

**Message:**

The shortened VMS-based screening tool can be a helpful instrument to identify frail older emergency department patients.

**Electronic supplementary material:**

The online version of this article (10.1007/s41999-020-00385-0) contains supplementary material, which is available to authorized users.

## Introduction

The global population is aging rapidly, which is associated with the increased use of emergency care by older people [1, 2]. Nowadays, older patients account for up to one-quarter of all emergency department (ED) attendances [3]. Research has demonstrated that older patients attending the ED are at risk of adverse events, which include unplanned hospitalization, return to the ED, and death [4, 5]. These adverse events are most likely due to a high prevalence of frailty, delayed diagnosis, and the presence of multiple comorbidities in older patients and can eventually result in permanent functional or cognitive decline [3, 6]. It is important to identify which patients are at risk for negative events, as several complications can be avoided using preventive interventions [7]. One approach to distinguish ED patients at risk is to screen for frailty [8, 9].

In the Netherlands, since 2012 all patients over 70 years who were admitted to the hospital are shortly screened for frailty using a screening instrument based on the national Safety Management System guidelines [*Veiligheidsmanagementsysteem* (VMS)] [6, 9]. The VMS for the vulnerable elderly is a Dutch risk assessment tool to identify older patients at risk of functional decline both during and after hospital admission. The instrument consists of thirteen questions regarding four important geriatric problems: delirium, falls, malnutrition, and physical impairment [6]. The instrument includes questions regarding memory problems, selfcare, a history of delirium or falls, either the Short Nutritional Assessment Questionnaire or Malnutrition Universal Screening Tool, and Katz Index of Independence in Activities of Daily Living-6. When a patient is at high risk to experience one or more geriatric problems, preventive interventions are introduced. Research showed that a high-risk score on multiple domains results in a higher risk of adverse events. However, it has not yet been validated for usage in the ED [10–13]. Screening tools validated for the ED include the Identification of Seniors at Risk (ISAR), Clinical Frailty Scale (CFS), and the recently introduced Dutch Acute Presenting Older Patient (APOP) instrument [14–16]. The ISAR and CFS instruments mainly focus on the functional and cognitive status of geriatric ED patients, however, several systematic reviews concluded that these tools are not able to predict adverse outcomes accurately [14, 17]. The APOP study developed a prediction model that included a wider variety of predictors, but the discrimination ability of the model was only fair [16]. Thus, there remains a need for a more accurate screening instrument to identify frail older ED patients. Since the VMS is originally developed for screening the risk for functional decline; it assesses domains that are associated with the incidence of frailty and highly correlate with functional decline, it may therefore better predict adverse outcomes in ED.

The University Medical Center Utrecht (UMCU), which is a 1042 bed tertiary academic teaching hospital in the Netherlands with approximately 20,500 annual ED visits, shortened the original VMS-based screening instrument to enhance its’ feasibility. This shortened version was implemented as part of the standard of care for older ED patients. This way, all patients are screened for vulnerability to adverse outcomes, and patients at risk are identified as early as possible. However, this shortened version had not been validated up to now. The aim of this study was to evaluate the prognostic value of the shortened version derived from the nationwide used VMS-based screening instrument for adverse outcomes in patients aged 70 years or older in the ED. First, the prognostic value of the shortened VMS-based screening instrument was evaluated for hospital admission, return ED visits within 30 days, and 90-day mortality. Further, the shortened tool was incorporated into a prediction model for 90-day mortality to improve predictive performance. This study aimed to identify which patients were at the highest risk of short-term mortality to early introduce preventive interventions.

## Methods

### Study population

A retrospective cohort study that included all patients aged 70 years or older who visited the ED of UMCU in November and December 2016 was conducted. In the Netherlands, patients are usually referred to the ED by a general practitioner, but self-referral, either by ambulance or own transport, is possible as well. Patients who were screened using the shortened VMS-based screening tool during ED visit were eligible for inclusion. Patients were excluded from analysis when the remark was made neither the patient nor its family was involved in completing the questionnaire or when it concerned scheduled ED visits due to non-emergency reasons. When patients visited the ED multiple times during the study period, only the first visit in which the VMS-score was correctly registered was included.

### VMS-based screening tool

The shortened screening instrument used at UMCU was derived from the original Dutch national VMS-based screening instrument and addressed the same four geriatric domains. The shortened version was implemented as part of the standard of care during an ED visit at UMCU and was in addition to the mandatory comprehensive VMS-based screening instrument for hospitalized older patients. The shortened tool consisted of five questions that concerned: (1) memory problems, (2) history of acute confusional state or delirium, (3) unintentional weight loss, (4) help in activities of daily living (ADL), and (5) falls in the past 6 months. This shortened VMS-based screening instrument was administered by an emergency department nurse during triage. The questions were answered with either ‘yes’ or ‘no’ by the patient or its’ family. The cumulative VMS-score was based on the sum of positive answers and ranged between 0 and 5. A VMS-score of ≥ 2 was arbitrarily chosen to be an increased risk for frailty and resulted in an automatic notification that advised the nurse to contact the geriatric department.

### Data collection

Data were retrieved from the electronic medical records. Baseline characteristics included age, sex, marital status, residency, comorbidities, use of medication, hospitalization in the prior 6 months, and mode of arrival to the ED. The residency was dichotomized into independent and dependent based on the availability of continuous care. The Charlson Comorbidity Index (CCI) was calculated for all patients [18]. Polypharmacy was defined as the use of ≥ 5 different types of medication. The adverse outcomes included hospital admission, return ED visits within 30 days, and 90-day mortality after ED visit. Only return ED visits at UMCU and deaths recorded in the patient’s medical record were registered.

### Statistical analysis

Descriptive analyses were presented as number and percentage for categorical data, as mean with standard deviation (SD) for normally distributed continuous data, and as median with interquartile range (IQR) for non-normally distributed continuous data. A Chi-square test was performed to assess the relationship between the dichotomized VMS-score (≥ 2) and outcomes. Univariate analyses were performed to assess potential confounders. We considered age, sex, marital status, residency, the CCI, polypharmacy, hospitalization in the prior 6 months, arrival by ambulance, and a high VMS-score (≥ 2) or the individual VMS-questions as potential confounders. All variables with *p* < 0.10 were included in multivariate analyses. The association between the VMS-score and hospitalization was assessed using multiple logistic regression and odds ratios (OR) with its 95% confidence interval (CI) were estimated. The prognostic value of the VMS-score for return ED visits and mortality was assessed using multiple Cox proportional hazard regression to estimate hazard ratios (HR) with its 95% CI.

Furthermore, a logistic regression model was developed to predict 90-day mortality. Candidate predictors included in a multivariate model were age, sex, marital status, residency, the CCI, polypharmacy, hospitalization in the prior 6 months, arrival by ambulance, and a high VMS-score (≥ 2). Non-significant variables were stepwise removed from the multivariate model using backward elimination. The significance level for variable exclusion was pre-defined as *p* > 0.10. Akaike’s Information Criterion (AIC) was used to identify the preferred candidate model, which was defined as the model with the lowest AIC value [19]. The total of predictor variables included in the final model were in compliance with the ten events per predictor variable (EPV) rule [20]. Discrimination was evaluated by determining the area under the receiver operating characteristic curve (AUC) and its 95% CI [19]. Internal validation was done by bootstrapping using 500 bootstrap samples. Every step of the model development was repeated for each bootstrap sample [19]. Afterwards, regression coefficients and AUC were adjusted to correct for optimism of model performance [21]. Adjusted predicted probabilities were calculated for each patient using the formula exp(*β*_0_ + *β*_1*x*_)/(1 + exp(*β*_0_ + *β*_1*x*_)). Calibration of the bias-corrected model was assessed by visual examination of the calibration slope and the Hosmer and Lemeshow test. Performance of the model was determined by calculating sensitivity, specificity, positive predictive value (PPV), negative predictive value (NPV), positive likelihood ratio (LR+), and negative likelihood ratio (LR−) with its 95% CI using different cut-off values based on the 10%, 20%, and 30% of the patients with the highest predicted probabilities. The optimal cut-off point was pre-defined as the threshold which resulted in a high negative predictive value and high sensitivity to minimize undertreatment.

Statistical analysis was performed using IBM SPSS Statistics for Windows, version 25.0 (IBM Corp., Armonk, N.Y., USA) and Stata Statistical Software, release 15 (StataCorp, College Station, TX, USA). *p* values < 0.05 were considered statistically significant.

## Results

In November and December 2016, a total of 752 ED visits were made by patients aged ≥ 70 years at UMCU. A VMS-score was administered during 542 visits (72.1%). After application of the exclusion criteria, 94 visits were excluded from analysis: 55 patients were already included during a previous ED visit, in 33 visits the screening was completed without the involvement of the patient or its’ caregiver, and 6 visits were scheduled visits due to non-emergency reasons. A flow chart was included in Supplementary Figure S1. In total, 448 patients were included in the analysis. Patient characteristics were presented in Table 1. The median age was 77 years (IQR 73–82) and 227 patients were male (50.7%). The median CCI was 5 (IQR 4–7). 115 patients (25.7%) had a VMS-score of ≥ 2.

**Table 1:** Patient characteristics at baseline

Characteristics	Patients (*n* = 448)
Age at ED visit in years	77 (IQR 73–82)
Male	227 (50.7%)
Married or partner	260 (58.0%)
Residence before ED visit	
Independent	218 (48.7%)
Dependent	35 (7,8%)
Unknown	195 (43.5%)
Charlson Comorbidity Index	5 (IQR 4–7)
Amount of medication	7 (IQR 4–11)
Hospitalization in the prior 6 months	155 (34.6%)
Transportation to ED by ambulance	269 (60.0%)
Short VMS-based screening tool	
Positive for memory problems	100 (22.3%)
Positive for history of delirium	63 (14.1%)
Positive for unintentional weight loss	67 (15.0%)
Positive for ADL dependency	126 (28.1%)
Positive for fall < 6 months	87 (19.4%)
Score of VMS-based screening tool	1 (IQR 0–2)
0	217 (48.4%)
1	116 (25.9%)
2	58 (12.9%)
3	26 (5.8%)
4	22 (4.9%)
5	9 (2.0%)

Data are presented as number (percentage) or median (interquartile range (IQR))

*ED* emergency department, *VMS* Veiligheidsmanagementsysteem (Safety Management System), *ADL* activities of daily living

A total of 266 patients (59.4%) were admitted to the hospital. After correction for potential confounders including age, hospitalization in prior 6 months, and arrival by ambulance, a high VMS-score (≥ 2) was associated with greater odds of hospital admission [OR 2.26 (95% CI 1.32–3.86)]. Multivariate analysis of the individual VMS-questions showed that both a history of delirium and ADL dependency were associated with hospitalization with ORs of 2.13 (95% CI 1.06–4.30) and 2.68 (95% CI 1.57–4.57), respectively.

Fifty-nine patients (13.2%) had returned to the ED within 30 days and 42 patients (9.4%) were lost to follow-up. 26 patients (5.8%), who died within 30 days and did not revisit the ED before their death, were excluded from further analysis. The median time between ED visit and return to the ED was 13 days (IQR 4–20). Multivariate analysis showed no significant association between either the VMS-score or individual VMS-questions and the risk of return ED visits.

After 90 days of follow-up, 58 (12.9%) patients had died after a median time of 22 days (IQR 9–45) and 41 (9.2%) patients were lost to follow-up. Survival analysis demonstrated that a VMS-score ≥ 2, after adjustment for confounders including dependent residency, the CCI, hospitalization in the prior 6 months, and arrival by ambulance, was associated with significantly lower overall survival [HR 2.48 (95% CI 1.31–4.71)]. Furthermore, multivariate analysis of the individual VMS-questions illustrated that a history of delirium and ADL dependency were associated with lower overall survival [HR 1.92 (95% CI 1.02–3.60) and 2.68 (95% CI 1.35–5.30), respectively].

A complete overview of univariate and multivariate analyses regarding hospitalization, ED revisits and mortality was included in Supplementary Tables S1–3.

### Prediction model

**Fig. 1:**
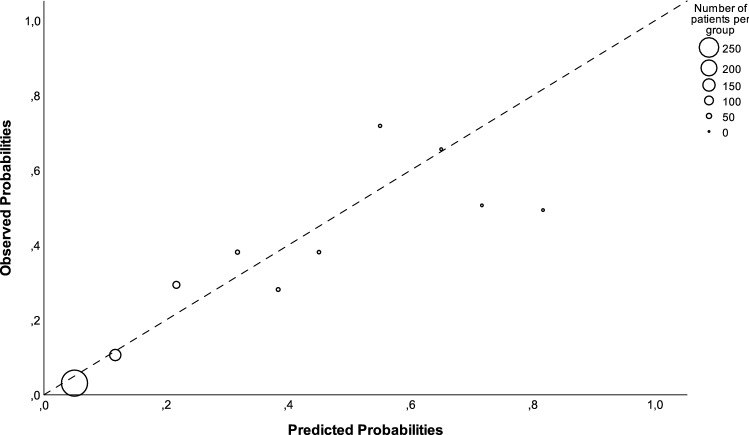
Calibration plot of the adjusted final model. ${\text{Adjusted predictedprobability}} = {\mkern 1mu} \frac{{e^{{\left( { - 4.279\, + \,'{\text{CCI'}}{\kern 1pt} \, \times \,{\kern 1pt} 0.280\, + \,\,'{\text{polypharmacy'}}\, \times \,{\kern 1pt} - \,0.901\, + \,'{\text{hospitalization prior }}6{\text{months'}}\, \times \,{\kern 1pt} 0.702\, + \,'{\text{arrival}}\,{\text{by ambulance'}}\, \times \,{\kern 1pt} 0.733\,\, + \,'{\text{VMS}} - {\text{score}} \ge 2'\,\, \times \,{\kern 1pt} 1.449} \right)}} }}{{1 + e^{{\left( { - 4.279\, + \,\,'{\text{CCI'}}\, \times \,{\kern 1pt} 0.280\, + \,'{\text{polypharmacy'}}\, \times \,{\kern 1pt} - \,0.901\, + \,'{\text{hospitalization}}\,{\text{prior }}6{\text{months'}}\,\, \times {\kern 1pt} 0.702\, + \,'\,{\text{arrival}}\,{\text{by ambulance'}}\, \times \,{\kern 1pt} 0.733\,\, + \,'{\text{VMS}} - {\text{score}} \ge 2'\, \times \,{\kern 1pt} 1.449} \right)}}}}$

A model was developed to predict 90-day mortality. After backward elimination, predictors included in the final model were the CCI, polypharmacy, hospitalization in the prior 6 months, arrival by ambulance, and a high VMS-score (≥ 2). An overview of the ORs and regression coefficients of the included predictors were provided in Supplementary Table S4. The final model was corrected for optimism using bootstrapping and had an AUC of 0.80 (95% CI 0.72–0.87). The formula to calculate the individual risk of each patient using the shrunken regression coefficients and its calibration plot are shown in Fig. 1. The calibration of the model was satisfactory with a Hosmer and Lemeshow Test of *p* = 0.727. The predictive performance of the model was evaluated and an overview for different cut-off points was given in Table 2. The preferred cut-off point was established at 30% of patients at the highest risk (which corresponded to a predicted probability of 0.143). This resulted in a sensitivity of 67.4%, a specificity of 75.3%, PPV of 28.5%, and NPV of 94.1%.

**Table 2:** Summary of the predictive performance of a model for 90-day mortality

Cut-off method (cut-off PP value)	Sensitivity (95% CI)	Specificity (95% CI)	PPV (95% CI)	NPV (95% CI)	LR+ (95% CI)	LR− (95% CI)
10% at highest risk (0.318)	36.7 (23.4–51.7)	93.8 (90.6–96.1)	46.2 (33.0–59.9)	91.0 (89.1–92.7)	5.9 (3.4–10.2)	0.7 (0.5–0.8)
20% at highest risk (0.206)	61.2 (46.2–74.8)	86.0 (81.8–89.5)	39.0 (31.1–47.4)	93.8 (91.4–95.6)	4.4 (3.1–6.2)	0.5 (0.3–0.6)
30% at highest risk (0.143)	67.4 (52.5–80.1)	75.3 (70.3–79.8)	28.5 (23.3–34.2)	94.1 (91.3–96.0)	2.7 (2.1–3.6)	0.4 (0.3–0.7)

*PP* predictive probability, *CI* confidence interval, *PPV* positive predictive value, *NPV* negative predictive value, *LR+* positive likelihood ratio, *LR−* negative likelihood ratio

## Discussion

Our research demonstrated that a high VMS-score, as well as the individual VMS-questions regarding the history of delirium and ADL dependency, were associated with both higher risks of hospital admission and 90-day mortality. A prediction model was developed based on the CCI, polypharmacy, hospitalization in the prior 6 months, arrival by ambulance, and a high VMS-score to predict 90-day mortality and showed satisfactory calibration and good discrimination (AUC 0.80). A cut-off point that selected 30% of patients at the highest risk resulted in a high negative predictive value and moderate sensitivity and specificity. Thus, the prediction model showed to be a helpful instrument to rule out patients with a low short-term mortality risk that are not in need of immediate preventive interventions.

Early identification of vulnerable older ED patients is important to prevent potential adverse events. Although several studies have been published evaluating the comprehensive VMS-screening instrument for hospitalized older patients, to our knowledge this is the first study to assess a shortened VMS-based tool for ED usage [6, 10–12, 22, 23]. A wide variety of screening tools has been developed for usage in the ED, but several systematic reviews concluded no tool is able to accurately identify the frail older patient yet [14, 17, 24]. Our study combined a shortened version of an existing screening instrument with several objective criteria (CCI, polypharmacy, hospitalization in the prior 6 months, and arrival by ambulance) aiming to accurately predict 90-day mortality. A comparable study performed in Danish and Australian EDs, which combined the Criteria for Screening and Triaging to Appropriate aLternative care (CriSTAL tool) with the CFS to predict mortality within 3 months, showed similar results with an AUC of 0.794 in the Danish cohort [25].

In the Netherlands, frequently used screening tools for ED usage include the ISAR–Hospitalized Patients (ISAR-HP) and APOP [26]. The ISAR-HP consists of questions regarding help with instrumental ADL, usage of walking aids, need of guidance during traveling, and no education after age 14. Discrimination for the composite outcome of functional decline or 90-day mortality was lower in comparison to our study with an AUC of 0.69 (95% CI 0.65–0.73) [27]. The APOP study developed and later optimized a prediction model which includes age, gender, arrival by ambulance, need of regular help, need help bathing or showering, hospitalization in the prior 6 months, and impaired cognition [16, 28]. These results are almost similar to our research with an AUC of 0.74 (95% CI 0.71–0.77) for 90-day mortality and for the 30% patients at highest risk sensitivity of 61%, specificity of 73%, PPV of 20% and NPV of 95%. However, the APOP study was conducted in a larger prospective cohort and externally validated, which led to more reliable results with smaller CIs. Nevertheless, the variation in the included predictors between both models might attribute to the observed differences in model performance as well, as our model did neither include age nor gender, but did take comorbidities into account.

We found some unexpected findings during our research. For example, no independent association was observed between the shortened VMS-score and ED revisits. These results could be explained due to the fact that there are multiple other factors that affect the risk of ED revisits, including age, male sex, polypharmacy, and cognitive impairment [29]. As these risk factors are not elaborately addressed in the VMS-questionnaire, this could partially explain why no independent association between the shortened VMS-score and ED revisits was observed. In addition, we were unable to determine whether patients had visited EDs of other hospitals during the follow-up period. This may have led to an underreporting of ED revisits. Another remarkable finding in our study was the fact that polypharmacy was seen as a protective factor for mortality in our prediction model, which is in contrast with previous research [30]. This might be explained due to overcorrection in the multivariate model or the fact that polypharmacy is associated with multimorbidity and subsequently results in more regular visits to a physician.

This research, however, is subject to several limitations. First, the retrospective study design presumably introduced a selection bias, since the VMS-score was administered in 72,1% of ED visits. It is likely ED nurses would omit the screening when it seemed inappropriate to them. For example, the screening was occasionally omitted in acutely ill patients who required immediate medical care, such as patients with a cerebrovascular accident or major trauma. Unfortunately, we were unable to collect the characteristics of patients whose VMS-scores were not registered to confirm this assumption. However, it is likely this bias affected the prevalence of our adverse outcomes and therefore the predictive performance of our model. Another limitation is the fact that all VMS-questions are somewhat subjective, especially the question regarding unintentional weight loss. Questions were kept simple to maintain its’ feasibility for ED usage, but more specific questions may decrease its’ subjectivity and produce more reliable results. Further, we conducted a single-center study at UMCU, which is an academic hospital that provides specialized care and, therefore, the study population might not reflect the average older ED patient. Another limitation that might have affected the results was the fact that a high VMS-scores resulted in an automatic notification that advised the ED nurse to contact the geriatric department due to an increased risk of frailty. The involvement of the geriatric department might have influenced the outcomes due to the introduction of preventive interventions. Supposedly, this would result in an underestimation of the risks of adverse events. To continue, we may have missed some ED revisits and deaths, since we only had access to the patients’ medical records at UMCU. Therefore, some patients were considered lost to follow-up. Lastly, in all likelihood, our prediction model is still too optimistic due to the lack of external validation. Thus, external validation is initiated to determine the performance of our prediction model outside our study population.

The shortened VMS-based screening instrument is a brief and feasible tool for ED usage that addresses four important geriatric domains. It has been used at the ED of UMCU for several years, aiming to identify vulnerable patients as early as possible. This study demonstrated that the shortened VMS-based tool is able to accurately predict adverse events including hospitalization and short-term mortality. Unlike the original comprehensive VMS-based screening instrument, which is used for hospitalized older patients only, the shortened version feasible for ED captures vulnerable patients who are not admitted as well. In addition, it promotes awareness of geriatric syndromes among patients and health care workers. It meets the Dutch government requirements, which state that health care facilities are obliged to screen all ED patients aged 70 years or older who are admitted to the hospital for delirium [31]. As the comprehensive VMS-based screening tool is used in all Dutch hospitals, a shortened version for ED usage is preferable to maintain consistency in screening instruments [32].

Our prediction model, which combined the VMS-score with several objective criteria, showed promising results comparable to other studies and the model will be further improved in future research. In clinical practice, the model can be incorporated as a separate section of the medical record. This way, risk scores can be easily calculated and an automatic notification can notify health care workers when a patient is considered at high risk. We advise considering the involvement of the geriatric department for high-risk patients to further determine whether and which preventive interventions are deemed appropriate for the individual patient. Examples of preventive interventions may include a Comprehensive Geriatric Assessment, the involvement of physiotherapist or dietitian, or delirium prevention.

To conclude, the shortened VMS-based screening instrument showed to be of good prognostic value for hospital admission and 90-day mortality and can be a helpful instrument for identifying frail older ED patients. The prediction model we developed for 90-day mortality showed promising results and will be further validated and optimized in future research.

## Electronic supplementary material

Below is the link to the electronic supplementary material.

Supplementary file1 (DOCX 66 kb)
