# Supplementary material for: Prognostic value of screening instrument based on the Dutch national VMS guidelines for older patients in the emergency department
Source: Eur Geriatr Med. 2020 Sep 1;12(1):143–50. doi: 10.1007/s41999-020-00385-0 (PMC7900072; doi:10.1007/s41999-020-00385-0)
Supplement: Supplementary file 1 — Supplementary file1 (DOCX 66 kb) [file 41999_2020_385_MOESM1_ESM.docx]

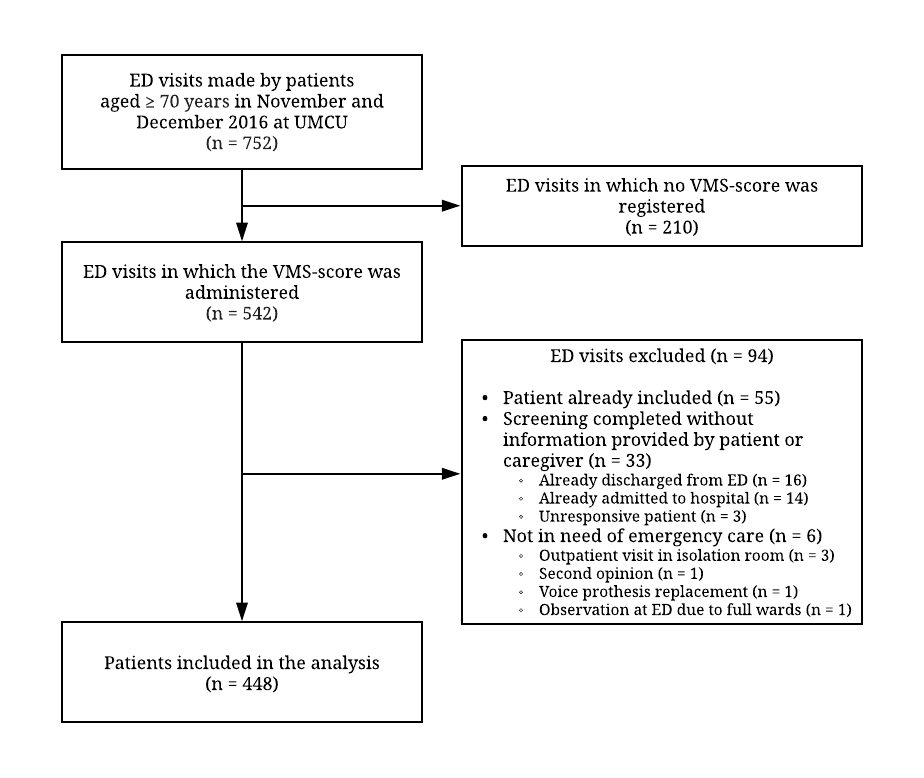


**Fig. S1** Flowchart of patient selection
*ED* emergency department, *UMCU* University Medical Center Utrecht, *VMS* Veiligheidsmanagementsysteem (Safety Management System)

**Table S1** Risk of hospitalization

|  | Univariate analysis | | Multivariate analysis | | | | | | | | | |
| --- | --- | --- | --- | --- | --- | --- | --- | --- | --- | --- | --- | --- |
| Variables | **OR (95% CI)** | **p-value** | **OR**  **(95% CI)** | **p-value** | **OR**  **(95% CI)** | **p-value** | **OR**  **(95% CI)** | **p-value** | **OR**  **(95% CI)** | **p-value** | **OR**  **(95% CI)** | **p-value** |
| Age | 1.04 (1.01 - 1.07) | 0.016 | 1.04 (1.00 - 1.07) | 0.102 | 1.04 (1.00 - 1.08) | 0.028 | 1.04 (1.00 - 1.08) | 0.029 | 1.03 (1.00 - 1.07) | 0.075 | 1.04 (1.01 - 1.08) | 0.018 |
| Sex† | 1.02 (0.70 - 1.49) | 0.925 |  |  |  |  |  |  |  |  |  |  |
| Living together | 1.37 (0.85 - 2.20) | 0.201 |  |  |  |  |  |  |  |  |  |  |
| Independent residency | 0.90 (0.41 - 1.97) | 0.784 |  |  |  |  |  |  |  |  |  |  |
| CCI | 1.06 (0.97 - 1.17) | 0.187 |  |  |  |  |  |  |  |  |  |  |
| Polypharmacy | 1.28 (0.83 - 1.97) | 0.271 |  |  |  |  |  |  |  |  |  |  |
| Hospitalization in the prior 6 months | 1.42 (0.94 - 2.15) | 0.096 | 1.60 (1.02 - 2.49) | 0.040 | 1.72 (1.11 - 2.67) | 0.016 | 1.58 (1.01 - 2.47) | 0.046 | 1.46 (0.92 - 2.29) | 0.106 | 1.74 (1.12 - 2.71) | 0.014 |
| Arrival by ambulance | 3.23 (2.17 -4.80) | 0.000 | 2.81 (1.83 - 4.32) | 0.000 | 2.99 (1.96 - 4.58) | 0.000 | 2.89 (1.89 - 4.43) | 0.000 | 2.71 (1.76 - 4.17) | 0.000 | 2.98 (1.95 - 4.56) | 0.000 |
| VMS-score ≥ 2 | 2.82 (1.74 - 4.57) | 0.000 | 2.26 (1.32 - 3.86) | 0.003 |  |  |  |  |  |  |  |  |
| *VMS-question memory problems* | *1.91 (1.18 - 3.09)* | *0.008* |  |  | *1.56 (0.91*  *- 2.67)* | *0.103* |  |  |  |  |  |  |
| *VMS-question history of confusion/delirium* | *2.69 (1.44 - 5.04)* | *0.002* |  |  |  |  | *2.13 (1.06 - 4.30)* | *0.034* |  |  |  |  |
| *VMS-question unintentional weight loss* | *1.59 (0.92 - 2.78)* | *0.100* |  |  |  |  |  |  |  |  |  |  |
| *VMS-question daily help in ADL* | *3.38 (2.10 - 5.46)* | *0.000* |  |  |  |  |  |  | *2.68 (1.57 - 4.57)* | *0.000* |  |  |
| *VMS-question falls in the past 6 months* | *1.77 (1.07 - 2.94)* | *0.026* |  |  |  |  |  |  |  |  | *1.59 (0.90 - 2.81)* | *0.109* |

*OR* odds ratio, *CI* confidence interval, *CCI* Charlson Comorbidity Index, *VMS* Veiligheidsmanagementsysteem (Safety Management System)

† Male as reference standard

**Table S2** Risk of ED revisit within 30 days

|  | Univariate analysis | |
| --- | --- | --- |
| Variables | **HR (95% CI)** | **p-value** |
| Age | 0.98 (0.94 - 1.03) | 0.466 |
| Sex† | 0.84 (0.50 - 1.40) | 0.505 |
| Living together | 0.98 (0.54 - 1.78) | 0.940 |
| Independent residency | 0.82 (0.29 - 2.32) | 0.712 |
| CCI | 1.04 (0.92 - 1.17) | 0.510 |
| Polypharmacy | 1.11 (0.59 - 2.09) | 0.755 |
| Hospitalization in the prior 6 months | 1.62 (0.97 - 2.71) | 0.067 |
| Arrival by ambulance | 0.92 (0.55 - 1.53) | 0.743 |
| VMS-score ≥ 2 | 0.80 (0.43 - 1.51) | 0.495 |
| *VMS-question memory problems* | *0.71 (0.35 - 1.44)* | *0.342* |
| *VMS-question history of confusion/delirium* | *1.05 (0.50 - 2.22)* | *0.890* |
| *VMS-question unintentional weight loss* | *0.85 (0.40 - 1.79)* | *0.672* |
| *VMS-question daily help in ADL* | *1.15 (0.65 - 2.02)* | *0.630* |
| *VMS-question falls in the past 6 months* | *0.80 (0.40 - 1.63)* | *0.546* |

*HR* hazard ratio, *CI* confidence interval, *CCI* Charlson Comorbidity Index, *VMS* Veiligheidsmanagementsysteem (Safety Management System)

† Male as reference standard

**Table S3** Risk of death within 90 days

|  | Univariate analysis | | Multivariate analysis | | | | | | | | | |
| --- | --- | --- | --- | --- | --- | --- | --- | --- | --- | --- | --- | --- |
| Variables | **HR (95% CI)** | **p-value** | **HR**  **(95% CI)** | **p-value** | **HR**  **(95% CI)** | **p-value** | **HR**  **(95% CI)** | **p-value** | **HR**  **(95% CI)** | **p-value** | **HR**  **(95% CI)** | **p-value** |
| Age | 1.00 (0.96 - 1.04) | 0.939 |  |  |  |  |  |  |  |  |  |  |
| Sex† | 0.74 (0.44 - 1.25) | 0.282 |  |  |  |  |  |  |  |  |  |  |
| Living together | 0.89 (0.48 - 1.64) | 0.696 |  |  |  |  |  |  |  |  |  |  |
| Independent residency | 2.47 (1.24 - 4.89) | 0.010 | 1.30 (0.64 - 2.64) | 0.477 | 1.41 (0.67 - 2.98) | 0.352 | 1.44 (0.71 - 2.91) | 0.312 | 1.64 (0.81 - 3.32) | 0.168 | 1.02 (0.48 - 2.17) | 0.953 |
| CCI | 1.30 (1.18 - 1.44) | 0.000 | 1.23 (1.09 - 1.38) | 0.001 | 1.26 (1.11 - 1.42) | 0.000 | 1.24 (1.10 - 1.39) | 0.001 | 1.24 (1.09 - 1.40) | 0.001 | 1.28 (1.13 - 1.45) | 0.000 |
| Polypharmacy | 1.01 (0.55 - 1.88) | 0.967 |  |  |  |  |  |  |  |  |  |  |
| Hospitalization in the prior 6 months | 2.16 (1.23 - 3.80) | 0.008 |  |  |  |  |  |  |  |  |  |  |
| Arrival by ambulance | 2.63 (1.42 - 4.88) | 0.002 | 3.42 (1.43 - 8.20) | 0.006 | 3.79 (1.59 - 9.07) | 0.003 | 3.67 (1.53 - 8.78) | 0.004 | 3.60 (1.50 - 8.62) | 0.004 | 3.51 (1.46 - 8.44) | 0.005 |
| VMS-score ≥ 2 | 4.07 (2.42 - 6.83) | 0.000 | 2.48 (1.31 - 4.71) | 0.005 |  |  |  |  |  |  |  |  |
| *VMS-question memory problems* | *2.24 (1.31 - 3.83)* | *0.003* |  |  | *1.39 (0.72*  *- 2.67)* | *0.323* |  |  |  |  |  |  |
| *VMS-question history of confusion/delirium* | *3.10 (1.78 - 5.42)* | *0.000* |  |  |  |  | *1.92 (1.02 - 3.60)* | *0.043* |  |  |  |  |
| *VMS-question unintentional weight loss* | *2.57 (1.46 - 4.52)* | *0.001* |  |  |  |  |  |  | *1.84 (0.96 - 3.52)* | *0.066* |  |  |
| *VMS-question daily help in ADL* | *4.40 (2.60 - 7.45)* | *0.000* |  |  |  |  |  |  |  |  | *2.68 (1.35 -5.30)* | *0.005* |
| *VMS-question falls in the past 6 months* | *1.39 (0.76 - 2.54)* | *0.279* |  |  |  |  |  |  |  |  |  |  |

*HR* hazard ratio, *CI* confidence interval, *CCI* Charlson Comorbidity Index, *VMS* Veiligheidsmanagementsysteem (Safety Management System)

† Male as reference standard

**Table S4** Predictors included in the final multivariate prediction model

| Predictor | β | OR (95% CI) | p-value | β_adj_ |
| --- | --- | --- | --- | --- |
| CCI | 0.293 | 1.34 (1.15 - 1.56) | 0.000 | 0.280 |
| Polypharmacy | -0.905 | 0.40 (0.17 - 0.99) | 0.047 | -0.901 |
| Hospitalization in the prior 6 months | 0.731 | 2.08 (1.03 - 4.17) | 0.040 | 0.702 |
| Arrival by ambulance | 0.742 | 2.10 (1.01 - 4.36) | 0.047 | 0.733 |
| VMS-score ≥ 2 | 1.490 | 4.44 (2.23 - 8.82) | 0.000 | 1.449 |

*β* regression coefficient, *βadj* regression coefficient adjusted after bootstrapping, *OR* odds ratio, *CI* confidence interval, *CCI* Charlson Comorbidity Index, *VMS* Veiligheidsmanagementsysteem (Safety Management System)
